# First visual record of rare purple-colored dogwhelks ( *Nucella lapillus*) on the Atlantic coast of Nova Scotia, Canada

**DOI:** 10.12688/f1000research.9707.2

**Published:** 2017-08-23

**Authors:** Sonja M. Ehlers, Julius A. Ellrich

**Affiliations:** 1 St. Francis Xavier University, Antigonish, Canada

**Keywords:** dogwhelk, Nucella lapillus, color, snail, rocky intertidal, rareness, temperature

## Abstract

The dogwhelk *Nucella lapillus* is a rocky intertidal gastropod of the North Atlantic coast. Individual shell color varies. Common colors range between white and brown, with darker dogwhelks being more affected by heat stress than lighter-colored conspecifics. Other reported shell colors are purple, black, mauve, pink, yellow, and orange from UK coasts, red and gray from the Bay of Fundy coast of New Brunswick and Nova Scotia (Canada), and purple, black, gray, yellow, and orange from the coasts of Maine and Massachusetts (USA), with purple being considered as a rare color. On the Atlantic coast of Nova Scotia, dogwhelks are active from April until November, but information on dogwhelk shell color is missing for this coast. On 16 June 2016, we found two purple-colored dogwhelks in the mid-to-high intertidal zone of a moderately wave-exposed rocky shore near Duncans Cove, on the Atlantic coast of Nova Scotia while collecting dogwhelks (n= 1000) during low tide for manipulative field experiments. All other dogwhelks collected on that day were of common white and brown colors. During earlier dogwhelk collections in Atlantic Nova Scotia (between 2011-2013) and field surveys in Duncans Cove (between 2014-2016), we did not find any purple-colored dogwhelks, indicating the rareness of this color in that region. Apparently, our observations provide the first visual record of rare purple-colored dogwhelks on the Atlantic coast of Nova Scotia, Canada.

## Introduction

The dogwhelk *Nucella lapillus* (L. 1758) is a common predatory gastropod in the rocky intertidal of the North Atlantic that feeds on barnacles and mussels ( Crothers, 1985; Etter, 2007). Individuals vary in shell color. White and brown are common colors ( Berry & Crothers, 1974; Crothers, 1983; Crothers, 1985; Etter, 1988). Other shell colors reported are purple, black, gray, mauve, pink, yellow, and orange on UK coasts ( Berry & Crothers, 1968; Berry & Crothers, 1974; Castle & Emery, 1981; Cooke, 1915; Moore, 1936), red and gray from the Bay of Fundy coast of New Brunswick and Nova Scotia (Canada) ( Colton, 1922; Crothers, 1983), and black, purple, gray, yellow, and orange from the coasts of Maine ( Colton, 1916; Colton, 1922; Crothers, 1983) and Massachusetts (USA) ( Etter, 1988). Purple is considered to be a rare color in dogwhelks ( Berry & Crothers, 1968; Colton, 1922; Etter, 1988). As shell color in the closely related dogwhelk *Nucella emarginata* is inherited ( Palmer, 1984; Palmer, 1985), a genetic control of the shell color has been suggested for *N. lapillus* ( Etter, 1988). Variation in shell color may protect the dogwhelks from visual predators ( Colton, 1916; Colton, 1922; Etter, 1988). Moreover, the occurrence of colored dogwhelks along the shore is influenced by physiological stress from heat and desiccation during tidal emersion periods, because darker-colored dogwhelks suffer more from desiccation than lighter-colored conspecifics as they show a higher energy intake from sunlight ( Etter, 1988; Harris & Jones, 1995). On the Atlantic coast of Nova Scotia, dogwhelks are active from April until November ( Hughes, 1972; Hunt & Scheibling, 1998), but information on dogwhelk shell colors is missing for this coast.

## Methods

On 16 June 2016, we collected 1000 dogwhelks along a 300 meter transect located in the mid-to-high intertidal of a moderately wave-exposed rocky coast with dense mussel ( *Mytilus* spp.) patches and seaweed ( *Fucus vesiculosus*) canopies near Duncans Cove (44°29’41.22”N, 63° 31’26.66”W), Halifax on the Atlantic coast of Nova Scotia. We collected the dogwhelks during low tide for manipulative field experiments to examine nonconsumptive effects (NCEs) of these predators on their prey. Equal dogwhelk quantities were collected by one of us (JAE) for related research projects on dogwhelk NCEs (e.g. Ellrich *et al.*, 2015; Ellrich *et al.*, 2016) in several locations, with similar levels of intertidal elevation and wave exposure, along the Atlantic coast of Nova Scotia: in Glasgow Head (45°19’12.61”N, 60°17’34.15”W) in May and June 2011, in Deming Island (45°12’44.31”N, 61°10’25.99”W) in May 2012, and in Deming Island, Halfway Cove (45°20’58.98”N, 61°21’46.58”W), and Half Island Cove (45°21’19.77”N, 61°11’23.73”W) in May and June 2013.

During field surveys for another research project near our dogwhelk collection site in Duncans Cove, dogwhelk colors were observed regularly during low tides (on 12 August 2014, 1 September 2015, and 21 August 2016). To observe dogwhelk colors, 30 quadrats (25 cm × 25 cm) along a 150 m transect parallel to the coastline were sampled at random on each survey date.

## Results & discussion

**Figure 1.**
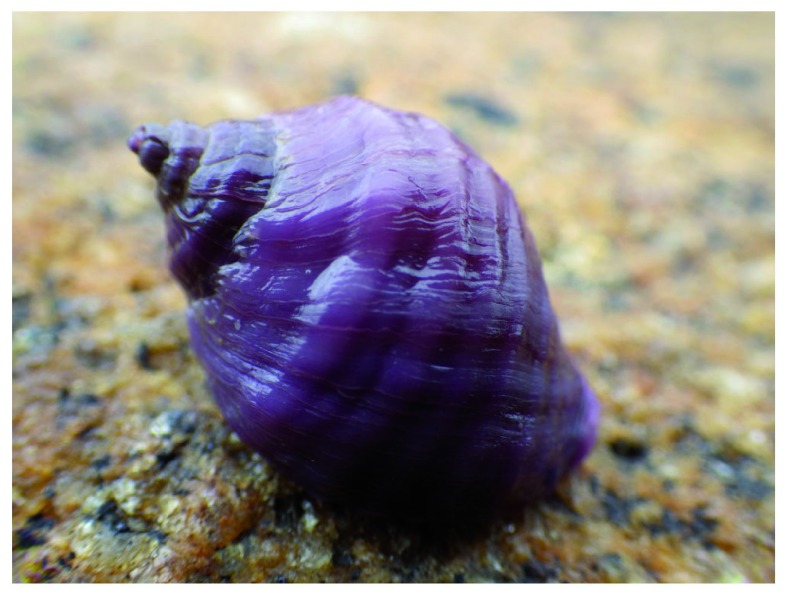
A purple-colored dogwhelk, *Nucella lapillus* (L. 1758). Picture taken near Duncans Cove (44°29’41.22”N, 63° 31’26.66”W), Halifax on the Atlantic coast of Nova Scotia, Canada on 16 June 2016 (picture credit: Julius A. Ellrich).

During our collection of dogwhelks near Duncans Cove on 16 June 2016 (n= 1000 dogwhelks in total), we found two dogwhelks of purple shell color. Our results provide the first visual record of purple-colored dogwhelks on the Atlantic coast of Nova Scotia ( Figure 1). The other dogwhelks collected on that day were of common brown and white shell colors. We did not find any other purple-colored dogwhelk during any of our five collections of equal dogwhelk quantities along the Atlantic Coast of Nova Scotia (n= 5000 dogwhelks of brown and white shell color in total) or three field surveys near Duncans Cove (n= 82 dogwhelks of brown and white shell color in total) indicating that purple-colored dogwhelks are rare in that region.

Previous observations along Massachusetts (USA) coasts found that darker-colored dogwhelks, including a small fraction of purple-colored individuals, occur mainly in wave-exposed habitats, presumably as occasional wave splash cools and moistens these organisms during low tide and, thereby, enables their persistence in such habitats ( Etter, 1988). Our study supports that notion, as the two purple-colored dogwhelks were found on a moderately wave-exposed coast. In addition, we found the two purple-colored dogwhelks next to dense mussel patches and seaweed ** canopies that retain moisture during low tide and, thereby, limit physiological stress from desiccation for intertidal organisms ( Beermann *et al.*, 2013; Etter, 1988). Hence, the occurrence of wave splash as well as the presence of mussel patches and seaweed canopies probably enhanced the chance of finding the rare purple-colored dogwhelks.

Future research could examine if dogwhelk behavioral responses to physiological stress from high temperatures vary with shell color. For example, purple-colored dogwhelks may find it less thermally stressful to venture out of crevices and macroalgal cover under relatively cool temperatures. Darker dogwhelks show stronger responses to heat, such as faster desiccation, than lighter-colored conspecifics ( Etter, 1988; Harris & Jones, 1995). Future experiments could, thus, examine if dogwhelk behavioral responses to temperature are related to shell color, which may contribute to the rareness in the observed purple-colored dogwhelks.

## References

[ref-1] Beermann AJ Ellrich JA Molis M: Effects of seaweed canopies and adult barnacles on barnacle recruitment: the interplay of positive and negative influences. *J Exp Mar Bio Ecol.* 2013;448:162–170. 10.1016/j.jembe.2013.07.001

[ref-2] Berry RJ Crothers JH: Stabilizing selection in the dog-whelk ( *Nucella lapillus*). *J Zool (Lond).* 1968;155:5–17. 10.1111/j.1469-7998.1968.tb03027.x

[ref-3] Berry RJ Crothers JH: Visible variation in the dog-whelk, *Nucella lapillus*. *J Zool (Lond).* 1974;174(1):123–148. 10.1111/j.1469-7998.1974.tb03147.x

[ref-4] Castle SL Emery AE: *Nucella lapillus*: a possible model for the study of genetic variation in natural populations. *Genetica.* 1981;56:11–15. 10.1007/BF00126924

[ref-5] Colton HS: On some varieties of *Thais lapillus* in the Mount Desert Region, a study of individual ecology. *Proc Acad Nat Sci Philadelphia.* 1916;68(3):440–454. Reference Source

[ref-6] Colton HS: Variation in the Dog Whelk, *Thais* ( *Purpura Auct.*) *lapillus.* *Ecology.* 1922;3(2):146–157. 10.2307/1929149

[ref-7] Cooke AH: The geographical distribution of *Purpura lapillus.* (L): Part I : In palæarctic waters *Journal of Molluscan Studies.* 1915;11(4):192–209. 10.1093/oxfordjournals.mollus.a063565

[ref-8] Crothers JH: Some observations on shell-shape variation in North American populations of *Nucella lapillus* (L.). *Biol J Linn Soc Lond.* 1983;19(3):237–274. 10.1111/j.1095-8312.1983.tb00786.x

[ref-9] Crothers JH: Dog-Whelks: an introduction to the biology of *Nucella lapillus* (L.). *Field Stud.* 1985;6:299–360. Reference Source

[ref-10] Ellrich JA Scrosati RA Molis M: Predator nonconsumptive effects on prey recruitment weaken with recruit density. *Ecology.* 2015;96(3):611–616. 10.1890/14-1856.1 26236858

[ref-11] Ellrich JA Scrosati RA Romoth K: Adult Prey Neutralizes Predator Nonconsumptive Limitation of Prey Recruitment. *PLoS One.* 2016;11(4):e0154572. 10.1371/journal.pone.0154572 27123994 PMC4849580

[ref-12] Etter RJ: Physiological stress and color polymorphism in the intertidal snail *Nucella lapillus*. *Evolution.* 1988;42(4):660–680. 10.1111/j.1558-5646.1988.tb02485.x 28563861

[ref-13] Etter R: Snails. In Denny, M. W., and S. D. Gaines. *Encyclopedia of Tidepools & Rocky Shores* University of California Press, Berkeley, California, USA,2007;530–537. Reference Source

[ref-14] Harris DJ Jones JS: Genotype-specific habitat selection and thermal ecology in *Nucella lapillus* (L.) (the dogwhelk). *Heredity.* 1995;74:311–314. 10.1038/hdy.1995.45

[ref-15] Hughes RN: Annual production of two Nova Scotian populations of *Nucella lapillus* (L.). *Oecologia.* 1972;8(4):356–370. 10.1007/BF00367538 28311257

[ref-16] Hunt HL Scheibling RE: Effects of whelk ( *Nucella lapillus* (L.)) predation on mussel ( *Mytilus trossulus* (Gould), *M. edulis* (L.)) assemblages in tidepools and on emergent rock on a wave-exposed rocky shore in Nova Scotia, Canada. *J Exp Mar Bio Ecol.* 1998;226(1):87–113. 10.1016/S0022-0981(97)00239-6

[ref-17] Moore HB: The biology of *Purpura lapillus.* I. Shell variation in relation to environment. *J Mar Biol Assoc U K.* 1936;21(1):61–89. 10.1017/S002531540001119X

[ref-18] Palmer AR: Species cohesiveness and genetic control of shell color and form in *Thais emarginata* (Prosobranchia, Muricacea): Preliminary results. *Malacologia.* 1984;25(2):477–491. Reference Source

[ref-19] Palmer AR: Genetic basis of shell variation in *Thais emarginata* (Prosobranchia, Muricacea). I. Banding in populations from Vancouver Island. *Biol Bull.* 1985;169(3):638–651. 10.2307/1541306

