# Supplementary material for: First visual record of rare purple-colored dogwhelks ( Nucella lapillus) on the Atlantic coast of Nova Scotia, Canada
Source: F1000Res. 2017 Aug 23;5:2435. Originally published 2016 Oct 4. [Version 2] doi: 10.12688/f1000research.9707.2 (PMC5580425; doi:10.12688/f1000research.9707.2)
Supplement: Peer review history

# Peer review: Referee response for version 2

**DOI:** 10.5256/f1000research.13449.r25322

**Published:** 2017-09-18

**Authors:** Mathieu Cusson

**Affiliations:** 1 Département des sciences fondamentales, Université du Québec à Chicoutimi (UQAC) , Chicoutimi, QC, Canada

The authors have addressed all comments and adjusted the paper accordingly. I recommend the acceptation of the revised paper. Nice piece of short communication!

I have read this submission. I believe that I have an appropriate level of expertise to confirm that it is of an acceptable scientific standard.

# Peer review: Referee response for version 2

**DOI:** 10.5256/f1000research.13449.r25320

**Published:** 2017-09-07

**Authors:** Jeff C. Clements

**Affiliations:** 1 Aquaculture and Coastal Ecosystems, Fisheries and Oceans Canada, Moncton, NB, Canada

The authors have done a great job with addressing my comments and I suggest that the paper be accepted for indexing. Nice work!

I have read this submission. I believe that I have an appropriate level of expertise to confirm that it is of an acceptable scientific standard.

# Peer review: Referee response for version 2

**DOI:** 10.5256/f1000research.13449.r25321

**Published:** 2017-08-29

**Authors:** Simon C. Courtenay

**Affiliations:** 1 School of Environment, Resources and Sustainability (SERS), Canadian Water Network (CWN), University of Waterloo, Waterloo, ON, Canada

The authors have adequately addressed my suggestions and I recommend that the paper now be accepted.

I have read this submission. I believe that I have an appropriate level of expertise to confirm that it is of an acceptable scientific standard.

# Peer review: Referee response for version 1

**DOI:** 10.5256/f1000research.10463.r16799

**Published:** 2016-12-01

**Authors:** Mathieu Cusson

**Affiliations:** 1 Département des sciences fondamentales, Université du Québec à Chicoutimi (UQAC) , Chicoutimi, QC, Canada

The authors report on a sighting of purple dogwhelks in Nova Scotia. During a sampling of over 1000 dogwhelks, they observed two purple-shelled individuals.

 Ehlers and Ellrich indicate that there may be a link between intertidal (air/water?) temperatures and the observation of the purple dogwhelks. A purple colouring is less thermally stressful in colder settings and due to the lower temperatures, the whelks may venture out from crevices and macroalgal cover.

 I work in the St. Lawrence Estuary where we have found dogwhelks in the intertidal zone although at lower quantities than those reported for Nova Scotia. Water and air temperatures are commonly under 12°C in this portion of the estuary, yet my team and I have never observed purple individuals - the typical colours for dogwhelks in this sector being white–beige to dark grey.

 The findings of Ehlers and Ellrich are of interest and it is worthwhile to pursue the subject with additional observations or experimental manipulations to understand the factors that affect shell colour variability. However, their linking of the purple colour to a single, cold day (lower water temperatures in the intertidal zone) as reported in this manuscript is rather anecdotic. Rather, the authors should list potential factors (both physiological and environmental) that could potentially affect shell coloration instead of linking this observation to a single day and the possible behaviour of two rare individuals. The potential link to behaviour - lower temperatures favouring an active movement of purple-shelled individuals out from shelter - is not convincing. Mauve coloration has been seen along European coasts. As this colour is very similar to purple, in which environmental conditions were these latter observations made? Similarly, if purple has also been reported from Maine, how would these observations from Maine confirm a link to colder temperatures? I suspect that the purple colour may simply be a rare phenotypic trait that may not be at all related to environmental factors (purple shells may be the albino individuals in dogwhelk populations?).

 Nevertheless, I do salute the authors for this valuable natural history observation that must be recorded. Thank you as well to F1000 for allowing the publication of such observations.  

 Other comments:

 Datasets: I do not understand why the data were separated into four distinct data sets, one for each date. I suggest a graph presenting all average temperatures coupled with the occurrence of white/brown and purple coloration. In the references, the authors list separate temperature data sets (one for each day). These data sources should be combined (not be separate) so as to avoid artificially increasing the number of publications.

 The observation date of June 16, the day on which the cool temperatures (12.3°C, n = 96) were recorded, is not presented in the companion data sets. Rather, I would prefer including a graph showing the recorded temperature for each relevant date.

I have read this submission. I believe that I have an appropriate level of expertise to confirm that it is of an acceptable scientific standard, however I have significant reservations, as outlined above.

# Response

**Published:** 2017-08-14

**Authors:** Julius Ellrich

**Affiliations:** St. Francis Xavier University, Canada

*The authors report on a sighting of purple dogwhelks in Nova Scotia. During a sampling of over 1000 dogwhelks, they observed two purple-shelled individuals.*

*Ehlers and *Ellrich *indicate that there may be a link between intertidal (air/water?) temperatures and the observation of the purple dogwhelks. A purple colouring is less thermally stressful in colder settings and due to the lower temperatures, the whelks may venture out from crevices and macroalgal cover.*

**

*I work in the St. Lawrence Estuary where we have found dogwhelks in the intertidal zone although at lower quantities than those reported for Nova Scotia. Water and air temperatures are commonly under 12°C in this portion of the estuary, yet my team and I have never observed purple individuals - the typical colours for dogwhelks in this sector being white–beige to dark grey.*

*The findings of Ehlers and* *Ellrich* *are of interest and it is worthwhile to pursue the subject with additional observations or experimental manipulations to understand the factors that affect shell colour variability. However, their linking of the purple colour to a single, cold day (lower water temperatures in the intertidal zone) as reported in this manuscript is rather anecdotic.*

*Rather, the authors should list potential factors (both physiological and environmental) that could potentially affect shell coloration instead of linking this observation to a single day and the possible behaviour of two rare individuals. The potential link to behaviour - lower temperatures favouring an active movement of purple-shelled individuals out from shelter - is not convincing. Mauve coloration has been seen along European coasts. As this colour is very similar to purple, in which environmental conditions were these latter observations made? Similarly, if purple has also been reported from Maine, how would these observations from Maine confirm a link to colder temperatures? I suspect that the purple colour may simply be a rare phenotypic trait that may not be at all related to environmental factors (purple shells may be the albino individuals in dogwhelk populations?).*

*Nevertheless, I do salute the authors for this valuable natural history observation that must be recorded. Thank you as well to F1000 for allowing the publication of such observations.*

 We appreciate the overall positive feedback on our manuscript and express our gratitude in the Acknowledgements. In the revised manuscript, we included the information that shell coloration in the dogwhelk *Nucella lapillus* is presumably inheritable. We agree that purple colored dogwhelks are rare. In addition, we discuss that their occurrence is likely favoured by the habitat characteristics (i.e. wave splash, presence of dense blue mussel beds and seaweed canopies) of the moderately wave-exposed coastline in Duncans Cove, Nova Scotia, which aligns closely with previous findings of rare purple colored dogwhelks along wave-exposed coasts in Massachusetts, USA (Etter 1988). Also, these habitat characteristics likely enhance the probability of finding rare purple colored dogwhelks.

 Etter, R. 1988. Physiological stress and color polymorphism in the intertidal snail *Nucella lapillus*. *Evolution*. 4(2): 660-680.

 ---

*Other comments:*

*Datasets: I do not understand why the data were separated into four distinct data sets, one for each date. I suggest a graph presenting all average temperatures coupled with the occurrence of white/brown and purple coloration. In the references, the authors list separate temperature data sets (one for each day). These data sources should be combined (not be separate) so as to avoid artificially increasing the number of publications.*

 In the revised manuscript, we removed the information on temperature in favor of information on wave-exposure as well as mussel bed and seaweed canopy presence. We did so, because we think that this information explains our findings of the two purple colored dogwhelks more conclusively.

**---**

*The observation date of June 16, the day on which the cool temperatures (12.3°C, n = 96) were recorded, is not presented in the companion data sets. Rather, I would prefer including a graph showing the recorded temperature for each relevant date.*

 The correct temperature data for 16 June 2016 was actually included, but unfortunately labeled wrong (i.e. 12 June 2016) in the companion data set. We apologize for the confusion. For the reasons discussed above, we removed the information on temperature from the revised manuscript.

 ---

# Peer review: Referee response for version 1

**DOI:** 10.5256/f1000research.10463.r16798

**Published:** 2016-11-01

**Authors:** Jeff C. Clements

**Affiliations:** 1 Aquaculture and Coastal Ecosystems, Fisheries and Oceans Canada, Moncton, NB, Canada

The article by Ehlers & Ellrich qualitatively documents the first record of the *Nucella lapillus* purple morph on the east coast of Canada. The authors also relate the observations of this colour morph to temperature, suggesting that cooler temperatures may allow this colour morph to become more active outside of rock crevasses.  

 I very much like this article. From my perspective, there are not enough outlets for basic biological observations such as this, which can serve as the basis for novel and important hypotheses. It is comforting to see F1000Research promoting observational records!

 I recommend that this observation be indexed once minor revisions are made to the article, which I highlight below:

1. The authors state that temperatures on the date of observation (16 June 2016) were cool relative to 3 other sampling dates. However, temperature data is not presented for 16 June (but is presented for 12 June). I would suspect that the temperature data for 12 June is not an accurate representation of those on 16 June. This can be rectified by including HOBO logger data for 16 June (if the authors have it) or by utilizing historical sea surface temperatures from online databases (e.g. DFO, NOAA, GoSL, etc.). 
2. Aside from mentioning that some colour morphs are more sensitive to temperatures than others, the authors do not provide much in the way of context for why this species exhibits such tremendous variation in shell colour. What is the ecological and evolutionary benefit (or hindrance) of having such variation in shell colour? A brief mention of this in the introduction and/or discussion would be useful. 
3. Although temperature means and their errors are reported in the abstract, I’m not a fan of downloading 4 separate Excel files of temperature data. I would suggest including a single graph of 24 h temperature recordings (or a bar chart of temperature means +- SE) for each sampling date. This will help the reader visualize the differences in temperature between the date when purple morphs were observed and the dates they were not.
4. I think the authors can add some more discussion points to the Results and Discussion section. Some specific suggestions are highlighted below:

- Some mention of the origin of purple morphs in eastern Canada would be nice. Do you think purple morphs have existed in eastern Canada for some time and were simply not observed until now? Or do you think this might be a consequence of ocean warming, with purple morphs moving to more northerly latitudes to avoid high temperatures? This is especially relevant given that the authors suggest that purple colour morphs do not fare well in higher temperatures.
- Some additional suggestions for future research are also warranted. For example, targeted sampling over a given time period in which temperatures vary might give a more quantitative understanding of *N. lapillus *colour morphs on the shores of Nova Scotia. Such a project would not only provide a quantitative description of *N. lapillus* colour morphs in eastern Canada (which is currently lacking), but could also inform on spatial and temporal overlaps in *N. lapillus* colour morphs and lend field evidence for behavioural responses to temperature (which would complement the authors’ suggestion of lab experiments well). Genetic testing could also provide evidence for the origin of these animals, advocating for or against a potential northward displacement in response to warming temperatures. 

I have read this submission. I believe that I have an appropriate level of expertise to confirm that it is of an acceptable scientific standard, however I have significant reservations, as outlined above.

# Response

**Published:** 2017-08-14

**Authors:** Julius Ellrich

**Affiliations:** St. Francis Xavier University, Canada

*The article by Ehlers & Ellrich qualitatively documents the first record of the Nucella lapillus purple morph on the east coast of Canada. The authors also relate the observations of this colour morph to temperature, suggesting that cooler temperatures may allow this color morph to become more active outside of rock crevasses.*

*I very much like this article. From my perspective, there are not enough outlets for basic biological observations such as this, which can serve as the basis for novel and important hypotheses. It is comforting to see F1000Research promoting observational records!*

*I recommend that this observation be published once minor revisions are made to the article, which I highlight below:*

We are pleased about the thoughtful assessment of our article and express our gratefulness in the Acknowledgements of our revised article. Below, we address the comments.

---

*1. The authors state that temperatures on the date of observation (16 June 2016) were cool relative to 3 other sampling dates. However, temperature data is not presented for 16 June (but is presented for 12 June). I would suspect that the temperature data for 12 June is not an accurate representation of those on 16 June. This can be rectified by including HOBO logger data for 16 June (if the authors have it) or by utilizing historical sea surface temperatures from online databases (e.g. DFO, NOAA, GoSL, etc.).*

The temperature data recorded by our HOBO loggers were actually from the dogwhelk collection day (i.e. 16 June 2016), but not from the 12 June 2016. When compiling the temperature data for the supplementary material of the manuscript, we accidentally labeled these data wrong. We are apologizing for the confusion. However, we excluded the temperature information from our revised manuscript in favor of information (i.e. wave-exposure, presence of mussel patches and seaweed canopies) that explains the finding of the two purple colored dogwhelks more conclusively.

---

*Aside from mentioning that some colour morphs are more sensitive to temperatures than others, the authors do not provide much in the way of context for why this species exhibits such tremendous variation in shell colour. What is the ecological and evolutionary benefit (or hindrance) of having such variation in shell color? A brief mention of this in the introduction and/or discussion would be useful.*

We included a brief statement on the consequences of shell color variation in the Introduction of the revised manuscript.

---

*3. Although temperature means and their errors are reported in the abstract, I’m not a fan of downloading 4 separate Excel files of temperature data. I would suggest including a single graph of 24 h temperature recordings (or a bar chart of temperature means +- SE) for each sampling date. This will help the reader visualize the differences in temperature between the date when purple morphs were observed and the dates they were not.*

In the revised manuscript, we replaced the temperature information by other environmental information as outlined above.

---

*4. I think the authors can add some more discussion points to the Results and Discussion section. Some specific suggestions are highlighted below:*

*i. Some mention of the origin of purple morphs in eastern Canada would be nice. Do you think purple morphs have existed in eastern Canada for some time and were simply not observed until now? Or do you think this might be a consequence of ocean warming, with purple morphs moving to more northerly latitudes to avoid high temperatures? This is especially relevant given that the authors suggest that purple color morphs do not fare well in higher temperatures.*

We think that purple colored dogwhelks have simply not been reported for the Atlantic coast of Nova Scotia yet, because they are relatively rare compared to the large quantities of the brown and white colored individuals commonly found on this coast. Purple colored individuals having moved to more northerly latitudes in response to ocean warming appears unlikely, because dogwhelks have a restricted activity range (which lies within tens of meters), and lack pelagic larval dispersal (Crothers 1985).

To add another discussion point to Results & Discussion in the revised manuscript, we included a paragraph that discusses the environmental influences (i.e. wave-exposure, presence of mussel patches and seaweed canopies) that may favor the occurrence of the rare purple colored dogwhelks on the coast.

Crothers, J. H. 1985. Dog-Whelks: an introduction to the biology of *Nucella lapillus* (L.). *Field Studies.* 6: 299-360.

**---**

*ii. Some additional suggestions for future research are also warranted. For example, targeted sampling over a given time period in which temperatures vary might give a more quantitative understanding of N. lapillus colour morphs on the shores of Nova Scotia. Such a project would not only provide a quantitative description of n. lapillus colour morphs in eastern Canada (which is currently lacking), but could also inform on spatial and temporal overlaps in N. lapillus colour morphs and lend field evidence for behavioural responses to temperature (which would complement the authors’ suggestion of lab experiments well). Genetic testing could also provide evidence for the origin of these animals, advocating for or against a potential northward displacement in response to warming temperatures.*

We suggested future research examining dogwhelk activity patterns in relation to shell color and temperature in the Results & Discussion of the revised manuscript. As discussed above, we deem that northward movement of dogwhelks in response to warming temperatures is unlikely.

---

# Peer review: Referee response for version 1

**DOI:** 10.5256/f1000research.10463.r16981

**Published:** 2016-10-14

**Authors:** Simon C. Courtenay

**Affiliations:** 1 School of Environment, Resources and Sustainability (SERS), Canadian Water Network (CWN), University of Waterloo, Waterloo, ON, Canada

This note reports, for the first time, the presence of a rare colour morph (purple) of the dogwhelk ( *Nucella lapillis*) near Halifax, Nova Scotia, Canada. This is an interesting observation which will be strengthened by two minor revisions to the presentation.

 First, it would be helpful in the Abstract and Methods to indicate that dogwhelks were (presumably) sampled during low tide when they were exposed to the air rather than in water.

 Secondly, temperature records are provided which would benefit from clarification. Judging from the range of temperatures, the temperature loggers were exposed to water during some of the tidal cycle and to air the rest of the time (i.e. they were placed in the intertidal). It would be helpful to know at what point in this temperature record the whelks were sampled. It might be that there are better measures of temperature to report than the daily average (and why do you report the sample size for temperatures recorded on one of your sample days (n=96) but not the other days? Should we assume the same n?).

 Also, the whelks were sampled on June 16, 2016 but the nearest temperature record is June 12, 2016. Yet the abstract talks about the temperature on the day of whelk sampling. Did I miss something?

 Finally, it would be useful to report whether you archived these unusual specimens, and perhaps some of the more usual colour morphs, in case a future researcher wants to look at them for possible morphological differences from the more common colour morphs. You note that there are physiological differences. With that said, I am delighted that there is a place that basic biological observations like these can still be reported. Well done F1000Research!

I have read this submission. I believe that I have an appropriate level of expertise to confirm that it is of an acceptable scientific standard, however I have significant reservations, as outlined above.

# Response

**Published:** 2017-08-14

**Authors:** Julius Ellrich

**Affiliations:** St. Francis Xavier University, Canada

*This note reports, for the first time, the presence of a rare colour morph (purple) of the dogwhelk (Nucella lapillus) near Halifax, Nova Scotia, Canada. This is an interesting observation which will be strengthened by two minor revisions to the presentation.*

 We appreciate the positive feedback on our report and express our gratitude in the Acknowledgements. We address each comment below.

***---***

**

*First, it would be helpful in the Abstract and Methods to indicate that dogwhelks were (presumably) sampled during low tide when they were exposed to the air rather than in water.*

 We added the information that dogwhelk collections and observations were done during low tide in the Abstract and Methods of the revised manuscript.

***---***

**

*Secondly, temperature records are provided which would benefit from clarification. Judging from the range of temperatures, the temperature loggers were exposed to water during some of the tidal cycle and to air the rest of the time (i.e. they were placed in the intertidal). It would be helpful to know at what point in this temperature record the whelks were sampled. It might be that there are better measures of temperature to report than the daily average (and why do you report the sample size for temperatures recorded on one of your sample days (n=96) but not the other days? Should we assume the same n?).*

*Also, the whelks were sampled on June 16, 2016 but the nearest temperature record is June 12, 2016. Yet the abstract talks about the temperature on the day of whelk sampling. Did I miss something?*

 As noted correctly by the reviewer, the temperature loggers recorded seawater temperature (while submerged) and air temperature (while emerged). The dogwhelks were collected/sampled within two hours around each low tide. The sample size of temperature recordings was identical for all days (n= 96 temperature recordings). The temperature recordings reported for the dogwhelk collection day were actually recorded by our temperature loggers on that day (i.e. 16 June 2016). Unfortunately, we labeled them wrong (i.e. 12 June 2016) when preparing the supplementary material for the manuscript. We apologize for the confusion. However, for the revised manuscript, we chose to replace the temperature information with other environmental information (i.e. wave-exposure and presence of dense mussel patches and seaweed canopies in Duncans Cove), as this information explains our findings of the purple colored dogwhelks more conclusively.

**---**

*Finally, it would be useful to report whether you archived these unusual specimens, and perhaps some of the more usual colour morphs, in case a future researcher wants to look at them for possible morphological differences from the more common colour morphs. You note that there are physiological differences. With that said, I am delighted that there is a place that basic biological observations like these can still be reported. Well done F1000Research!*

 Having collected thousands of dogwhelks in Nova Scotia over the years for manipulative experiments, we were very surprised when we discovered the two purple dogwhelks. We, thus, considered them as rare, and decided not to collect them. Instead, we took the picture (Fig. 1) to document our findings. However, we agree that archiving a collection of dogwhelk color morphs including purple individuals could be useful for future comparative studies. As we will continue our research on nonconsumptive predator effects using dogwhelks, we will take this opportunity to deposit a collection of dogwhelk color morphs in a zoological collection.

 ---
